# Environmental Enrichment From Birth Impacts Parvalbumin Expressing Cells and Wisteria Floribunda Agglutinin Labelled Peri-Neuronal Nets Within the Developing Murine Striatum

**DOI:** 10.3389/fnana.2019.00090

**Published:** 2019-10-24

**Authors:** Angela May O’Connor, Thomas Joseph Burton, Hannan Mansuri, Gabriel Rhys Hand, Catherine Anne Leamey, Atomu Sawatari

**Affiliations:** Systems Neuroscience Laboratory, Discipline of Physiology, School of Medical Sciences and the Bosch Institute, Faculty of Medicine and Health, The University of Sydney, Sydney, NSW, Australia

**Keywords:** perineuronal nets (PNNs), parvalbumin cell, development, striatum, basal ganglia, environmental enrichment (EE), BDNF

## Abstract

Environmental enrichment can dramatically affect both the development and function of neural circuits. This is accomplished, at least in part, by the regulation of inhibitory cellular networks and related extracellular matrix glycoprotein structures known as perineuronal nets. The degree to which enhanced housing can influence brain areas involved in the planning and execution of actions is not well known. We examined the effect of enriching mice from birth on parvalbumin expression and perineuronal net formation in developing and adult striatum. This input nucleus of the basal ganglia consists of topographically discernible regions that serve different functions, providing a means of simultaneously examining the influence of environmental factors on discrete, but related networks. Greater densities of striatal parvalbumin positive cells and wisteria floribunda agglutinin labelled perineuronal nets were present in enriched pups during the second postnatal week, primarily within the lateral portion of the nucleus. Housing conditions continued to have an impact into adulthood, with enriched mice exhibiting higher parvalbumin positive cell densities in both medial and lateral striatum. Curiously, no differences due to housing conditions were detected in striatal perineuronal net densities of mature animals. The degree of overlap between striatal parvalbumin expression and perineuronal net formation was also increased, suggesting that heightened neural activity associated with enrichment may have contributed to greater engagement of networks affiliated with cells that express the calcium binding protein. Brain derived neurotrophic factor, an important regulator of inhibitory network maturation, is also subtly, but significantly affected within the striatum of enriched cohorts. Together, these findings suggest that environmental enrichment can exert cell specific effects within different divisions of an area vital for the regulation of action.

## Introduction

Environmental enrichment (EE) is a useful means of revealing the influence of extrinsic factors on the development and function of neural networks (Faherty et al., 2003; Li et al., 2006; Simonetti et al., 2009). EE can have a profound influence throughout the life of an animal, including within the first few postnatal weeks (Cancedda et al., 2004), when circuits involved in assessing and acting on an animal’s surroundings exhibit heightened plasticity (i.e., a ‘critical’ or ‘sensitive’ period; Woolsey and Wann, 1976; Gordon and Stryker, 1996; Pizzorusso et al., 2002), as well as later in life (Sale et al., 2007).

The anatomical and functional effects of enrichment have been explored in visual cortex (Cancedda et al., 2004), as well as in other circuits (van Praag et al., 1999; Spires et al., 2004; Simonetti et al., 2009). Less is known about the influence of EE in the striatum, the input nucleus of the basal ganglia involved in regulating motor as well as goal-directed learning. Circuitry contributing to sensorimotor coordination and habit formation (lateral portion) is topographically separate from circuits involved in the consolidation of action-outcome associations (medial region) (Yin et al., 2004, 2006). These features make the striatum an ideal brain region for exploring the influence of environmental factors on the development of differentiated networks relevant to action.

The timing of developmental critical periods is dependent on the maturation of inhibitory circuits that express the calcium binding protein parvalbumin (PV) (Huang et al., 1999; Fagiolini and Hensch, 2000). Neurotrophins such as brain derived neurotrophic factor (BDNF) contribute to the initiation of PV network maturation (Huang et al., 1999), while the maintenance of a mature, relatively stable circuit depends on the formation of perineuronal nets (PNNs), extracellular matrix structures consisting of chondroitin sulfate proteoglycans that encapsulate PV cells (Hockfield et al., 1990; Hartig et al., 1994; Bruckner et al., 2000; McRae et al., 2007).

Striatal PV expression emerges between the first and third postnatal weeks (Plotkin et al., 2005; Lee et al., 2012) following a lateral to medial progression (Schlosser et al., 1999), coincident with a number of relevant developmental events, including the onset of coordinated motor behaviour (Schapiro et al., 1970; Simonetti et al., 2009). These regulators of striatal output are recipients of direct cortical and dopaminergic input, and exhibit a high lateral, low medial presence within the adult nucleus (Schlosser et al., 1999; Lee et al., 2012). Whether this developmental trajectory or the maintenance of PV expression is altered by early EE has yet to be determined.

PV cells have been shown to exhibit different expression levels of the calcium binding protein; interestingly, this is dependent on functional context (Donato et al., 2013, 2015). Increases in the proportion of cells expressing high levels of PV (Hi PV) reflect rule consolidation, while cells with low PV expression (Lo PV) become prominent during information acquisition (Donato et al., 2015). EE can influence the relative presence of these cell types, increasing the proportion of Lo PV cells within the hippocampus (Donato et al., 2013). While both populations are present in the striatum (Donato et al., 2015), it is not known whether the developmental progression and/or relative localisation of PV cell types are also altered by early EE.

The emergence of wisteria floribunda agglutinin (WFA) labelled PNNs exhibits a similar time-course to PV expression, with formation initiating between the second and third postnatal weeks within lateral striatum (Lee et al., 2008). Curiously, unlike PV cells, a greater density of these structures are present in the medial portion of the striatum in adult animals (Lee et al., 2012). Moreover, only around half of all striatal PV cells are encapsulated by PNNs (Lee et al., 2012). While early exposure to EE can accelerate the formation of these extracellular matrix structures (Simonetti et al., 2009), whether it further affects the degree of PV cell/PNN overlap is not clear.

Accordingly, the influence of early EE on the maturation of Hi and Lo PV cells was examined quantitatively at two ages in the murine striatum: postnatal day (P)15 corresponding to the emergence of striatal PV expression (Schlosser et al., 1999), and as adults (12 to 14 weeks of age; mature circuitry). EE from birth led to measurable increases in Lo PV densities in lateral striatum first at P15, with detectably higher concentrations of the same cell type observed in both medial and lateral regions in adult samples. EE also affected striatal PNN formation, with higher densities observed in enriched pups at P15, but not in adults. PV encapsulation by PNNs showed EE induced increases similar to PV expression. Striatal BDNF protein levels exhibited an increase just prior to the emergence of PV expression, along with a detectable change due to EE, suggesting a possible mechanistic link. Together, these findings indicate that enrichment affects the maturation and maintenance of functional circuitry within striatal divisions.

## Materials and Methods

### Ethics Statement

All procedures were approved by the Animal Ethics Committee of the University of Sydney and conformed to National Health and Medical Research Council of Australia guidelines. Experiments were performed on both male and female C57/BL6J mice which were purchased [Animal Resources Centre (ARC), Canning Vale, Western Australia] and reared at the University of Sydney Laboratory Animal Facilities. All mice were housed in a single adequately ventilated room in 21°C ambient temperature on a 12-h light-dark cycle with *ad libitum* access to dry food and water.

### Housing of Animals in Standard and Enriched Environments

Upon arrival in the colony, late-pregnant dams were housed in either standard (SE) or enriched (EE) (Simonetti et al., 2009) laboratory environments. SE cages (28 × 44 × 26 cm) contained one red mouse igloo, one paper roll and some added tissue for nesting material, and housed only one dam and her litter. EE cages (44 × 60 × 30 cm) contained two red mouse igloos, one running disk mounted at an angle on one of the igloos, and several additional enrichment objects (one ladder, six scented fluffy balls, four unscented fluffy balls, one plastic bell-ball, one shape made from two coloured pipe-cleaners, several loose ice pop sticks, and two high-contrast visual stimuli taped to the outside of the cage). These objects were chosen in order to stimulate as many senses as possible; the running disc provided access to voluntary exercise. The positions of enrichment objects in the EE cages were changed every 2 days for added stimulation and replaced/re-scented as required. Two dams and their litters were housed together in one enrichment cage, to provide increased opportunity for social interactions.

At 21 days postnatal (P21), pups were weaned and placed into sex-segregated housing of the same environmental conditions in which they were born.

### Measuring PV Cell Number and Overlap With PNNs in Fixed Tissue

P15 (4 pups per group for P15) and adult (12–14 weeks of age, 3 animals per group) mice were euthanised with >100 mg/kg of sodium pentobarbitone injected intraperitoneally, and transcardially perfused using 0.9% saline followed by 4% paraformaldehyde in 0.1M phosphate buffer (Dauth et al., 2016). Brains were removed, post-fixed overnight in 4% paraformaldehyde in 0.1M PB, and cryoprotected in 30% sucrose in 0.1M PB. Brains were then embedded in gelatin-albumin hardened by addition of a few drops 25% glutaraldehyde, and sectioned coronally at 60 μm on a freezing microtome.

Double-staining for parvalbumin expressing (PV) neurons, and perineuronal nets (PNNs) was accomplished by combining and adjusting published protocols (Fukuda and Kosaka, 2003; Fukuda, 2009; Lee et al., 2012). Sections from rostral to mid striatum [bregma 1.70 mm to bregma 0.50 mm (George and Franklin, 2004)] were included in the analysis. For PV staining, sections were cryoprotected in 30% sucrose 0.1M PB and put through a rapid freeze-thaw procedure using liquid nitrogen (Fukuda and Kosaka, 2003; Fukuda, 2009). Sections were then labelled for chondroitin-sulphate proteoglycans (CSPGs) using the plant lectin Wisteria Floribunda Agglutinin (WFA; Vector Labs, Burlingame, CA, United States), as described previously (Lee et al., 2012). Following this, sections were incubated 48–72 h at 4°C in a rabbit polyclonal antibody against parvalbumin [dilution 1:500; Abcam (catalogue number: ab11427; Antibody Registry ID: AB_298032), Cambridge, United Kingdom] (Akgul and Wollmuth, 2010; Bekku et al., 2012; McKenna et al., 2013), followed by 3 h of incubation in goat anti-rabbit AlexaFluor 594 (dilution 1:200; Life Technologies, New York, NY, United States), then mounted in 50/50 glycerol-0.1M PB with 1:1000 DAPI. Sections were digitally imaged at low power using a Zeiss deconvolution microscope with AxioCamHR camera and Axiovision software (Carl Zeiss Microscopy GmbH, Jena, Germany) and at high power using a Zeiss LSM 510 META confocal laser scanning microscope and Zeiss LSM software (Carl Zeiss Microscopy GmbH, Jena, Germany).

Counting of immuno- and lectin labelled elements was performed using Image J (Rasband, W.S., U. S. National Institutes of Health, Bethesda, MD, United States). The location of PNNs and PV cells were manually marked by investigators blind to housing condition. PVs, PNN, as well as PV/PNN overlap counts were obtained. Cells that exhibited the highest level (>75% of maximum intensity) of PV labelling after thresholding were defined as Hi PV (those that fell below this threshold were categorised as Lo PV cells). Both Hi and Lo PV populations were marked separately, blind to housing condition and age.

Medial and lateral striatal halves were defined as follows: for each section the medial-lateral midpoint defined at the maximal width of the nucleus was determined. A line parallel to the mid-sagittal was drawn through this midpoint. The area spanning the medial border of the striatum to this dividing line was considered “medial,” and the region bound by the same line to the lateral border as “lateral.”

Measurements were imported into SPSS (SPSS Inc., Chicago, IL, United States) for statistical analysis. Mixed model (MM) ANOVAs (age, and housing condition as between-subject factors; PV cell type, and regions as within-subject factors) were used to compare PV, PNN, and PV/PNN overlap densities [counts/μm^2^ (measured area of relevant striatal halves)]. Bonferroni corrected pair-wise comparisons were used to identify specific differences.

### Fresh Tissue and Protein Analysis

P8, P10, and P15 standard and enriched mice (*n* = 5 at P8; *n* = 8 at P10 and P15 for each housing group) were euthanised with >100 mg/kg of sodium pentobarbitone injected intraperitoneally. Animals were then decapitated and the brain immediately removed and placed on ice. The striatum (caudate and putamen nuclei) was rapidly dissected out of thick coronal slices spanning the anterior tip of the nucleus to the front end of the fornix and anterior commissure (portions of the nucleus as close as possible to the regions analysed anatomically), using ice-cold instruments and a dissection microscope as described previously (Chiu et al., 2007). All care was taken to avoid including other brain structures or white matter from the corpus callosum. All samples were weighed, then snap-frozen in liquid nitrogen and stored at −80°C until processed.

Striatal tissue was suspended in 1 mL of lysis buffer [137 mM sodium chloride (NaCl); 20 mM Tris-hydrogen chloride (Tris-HCl) (pH 8.0); 1% triton-x 100; 10% glycerol; 1 mM phenylmethylsulfonyl fluoride (PMSF); 10 μg/mL aprotonin; 1 μg/mL leupeptin; 0.5 mM sodium vanadate]. Samples were then sonicated, vortexed, and centrifuged at 1500 *g* for 20 min at room temperature. The concentration of BDNF was determined using the E-max ImmunoAssay system (Promega, Madison, WI, United States). Hundred μl of sample was added to each well in triplicate and the absorbance read at A450 (BMG POLARstar Galaxy microplate reader; MTX Lab Systems, Bradenton, VA, United States).

A univariate, multifactorial ANOVA was used to assess protein levels across age groups, using age and housing condition as between-subjects factors. Bonferroni corrected pair-wise comparisons were run to identify specific differences.

A significance value of α = 0.05 was assumed for all statistical tests.

## Results

### Environmental Enrichment Leads to Age Related, Region and Cell Type Specific Changes in Striatal PV Cell Populations

While experience and environmental factors are capable of modulating PV expression within hippocampus (Donato et al., 2013) and other areas (Ciucci et al., 2007), the degree to which enrichment from birth can affect striatal PV levels in developing and mature mice is not known.

**FIGURE 1:**
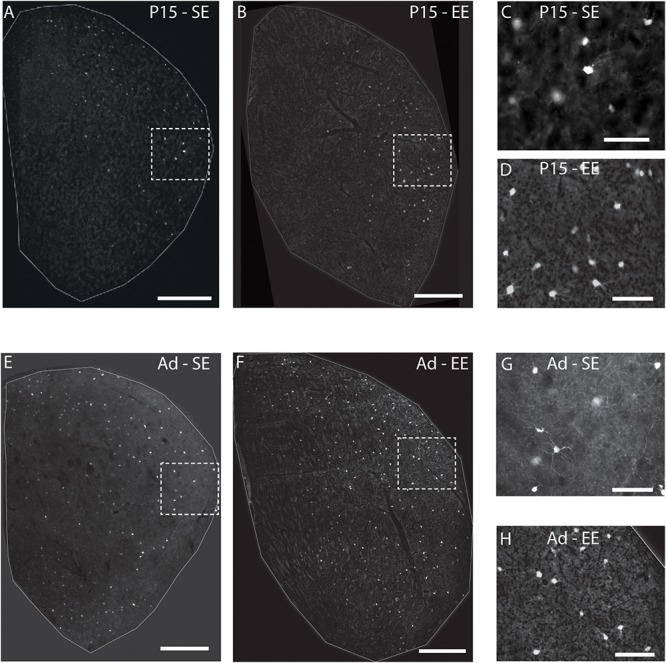
Environmental enrichment affects the emergence of striatal PV expression. Sample striatal sections showing the pattern of PV immunostaining (bright label) at P15 and adult samples of standard (SE) housed mice (**A,E** respectively). PV staining at approximately 2 weeks is most prominent in the lateral part of the striatum, spreading medially with age. Comparable sections depicting sample striatum of P15 and adult enriched (EE) mice (**B,F** respectively). Note the greater density of positively stained PV cells at both ages assessed compared to standard-housed controls at the same age. The rightmost, smaller, vertical pairs of panels depict higher power images of PV labelling from the white dotted line boxes in P15 (**C,D** for boxes in **A,B** respectively) and adult (**G,H** for **E,F** respectively) samples of the same row. Scale bars (in images): 350 μm in **(A,B,E,F)**; 100 μm for **(C,D,G,H)**.

Qualitative analysis of emergent PV expression suggested several changes in SE and EE cohorts between P15 and adulthood (Figure 1). Most of the differences seemed to initiate in the lateral half of the striatum. At P15, Hi as well as Lo PV cells were detectable primarily in the lateral striatum of SE and EE samples (Figures 1A,B; higher power images of areas outlined by the white boxes within the lateral striatum are shown in Figures 1C,D respectively). Both PV cell types continued to increase into adulthood for both housing groups (Figures 1E,F; higher power images of white boxed areas shown in Figures 1G,H respectively). Overall expression appeared to remain more prevalent within the lateral striatum, although clear PV labelling can be seen in the medial region in samples regardless of housing conditions. Notably, PV cells appeared to be more prevalent in EE versus SE samples at both ages examined.

Quantitative assessment supported these observations. When considering the effect of housing (SE vs. EE), age (P15 and adult), PV cell type (Hi vs. Lo), and topographic location (lateral vs. medial striatum), omnibus testing confirmed that PV cell densities were significantly affected by all factors [MM ANOVA, between subject main factor of housing condition: *F*(1,45) = 27.718, *p* < 0.001; age: *F*(1,45) = 204.707, *p* < 0.001; within subject factors for cell type: *F*(1,45) = 82.191, *p* < 0.001; and striatal region: *F*(1,45) = 480.385, p < 0.001]. A significant interaction was also detected, suggesting that housing conditions had a differential effect on PV populations depending on age, PV cell type, and striatal region [housing ^∗^ age ^∗^ cell type ^∗^ region: *F*(1,45) = 5.167, *p* = 0.028].

**FIGURE 2:**
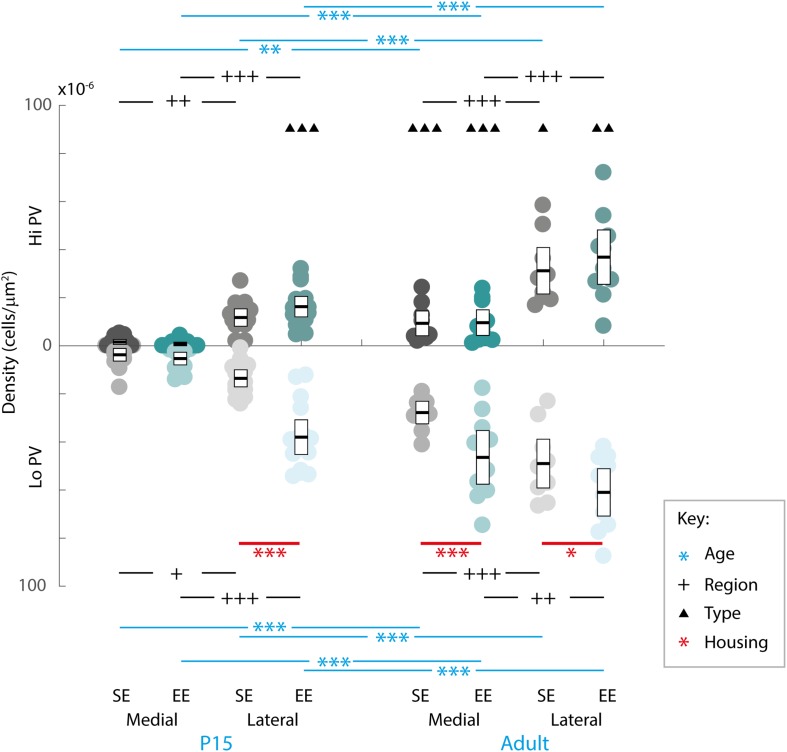
Age, region, cell type as well as housing conditions influence striatal PV expression. Striatal PV cell density (counts per micrometer^2^) in standard (SE) and enriched (EE) mice at all age points assessed. Blue bars and asterisks (Age): Both Hi PV (above abscissa) and Lo PV (below abscissa) cell densities were significantly greater in adult compared to P15 samples in SE medial (Hi PV: *p* = 0.001; Lo PV: *p* < 0.001), lateral (Hi PV: *p* < 0.001; Lo PV: *p* < 0.001), as well as EE medial (Hi PV: *p* < 0.001; Lo PV: *p* < 0.001) and lateral (Hi PV: *p* < 0.001; Lo PV: *p* < 0.001) striatum. Black bars and crosses (Regions): PV positive cell density in the lateral region was significantly higher compared to medial striatum for Hi and Lo PV cells in P15 SE (Hi PV: *p* = 0.001; Lo PV: *p* = 0.011), EE (Hi PV: *p* < 0.001; Lo PV: *p* < 0.001), as well as adult SE (Hi PV: *p* < 0.001; Lo PV: *p* < 0.001) and EE (Hi PV: *p* < 0.001; Lo PV: *p* = 0.002) samples. Black triangles (Hi versus Lo PV): While no differences between PV cell types were revealed for either region at P15 for SE mice (*p* > 0.398, all comparisons), significantly greater Lo PV compared to Hi PV densities were observed in lateral striatum for EE cohorts (*p* < 0.001). By adulthood, Lo PV densities were significantly higher in both medial and lateral portions of the nucleus for SE (SE medial: *p* < 0.001; lateral: *p* = 0.014) as well as EE samples (EE medial: *p* < 0.001; lateral: *p* = 0.001). Red bars and asterisks (Housing): At P15, significantly greater Lo PV densities were detected in EE relative to SE samples in lateral striatum (*p* < 0.001). Lo PV cells continued to exhibit significantly higher densities in both medial (*p* < 0.001) and lateral (*p* = 0.049) regions of adult EE sections. No differences in Hi PV densities were observed. Means (solid bar) and standard errors of the mean (s.e.m.; extent of white boxes) are depicted. Individual values are depicted as coloured circles: darker greys: medial SE; darker cyans: medial EE; lighter greys: lateral SE; Lighter cyans: lateral EE. ^∗^*p* < 0.05; ^∗∗^*p* < 0.01; ^∗∗∗^*p* < 0.001; ^+^ *p* < 0.05; ^++^*p* < 0.01; ^+++^*p* < 0.001; ^▲^*p* < 0.05; ^▲▲^*p* < 0.01; ^▲▲▲^*p* < 0.001. Key indicates parameter over which significance was found.

In order to gain a better appreciation of how each of the factors contributed to changes in PV expression, pairwise comparisons were examined across age, striatal region, cell type and housing conditions. With respect to age, the density of both Hi and Lo PV cells showed significant increases between P15 and 60 for SE medial (Hi PV: Adult > P15, *p* = 0.001; Lo PV: Adult > P15, *p* < 0.001), lateral (Hi PV: Adult > P15: *p* < 0.001; Lo PV: Adult > P15: *p* < 0.001), as well as EE medial (Hi PV: Adult > P15, *p* < 0.001; Lo PV: Adult > P15, *p* < 0.001) and lateral (Hi PV: Adult > P15: *p* < 0.001; Lo PV: Adult > P15: *p* < 0.001) striatum [relevant descriptive statistics can be found in Table 1; Figure 2, blue lines with asterisks indicate significance levels across relevant age related comparisons (same conventions used for subsequent figures)].

**TABLE 1:** PV cell densities.

**Age**	**Housing**	**Type**	**Region**	**Mean**	**Standard Error**
P15	SE	Hi PV	Medial	1.593	1.313
			Lateral	11.746	3.032
		Lo PV	Medial	3.750	2.407
			Lateral	13.531	3.324
	EE	Hi PV	Medial	0.756	1.313
			Lateral	16.282	3.032
		Lo PV	Medial	5.318	2.407
			Lateral	38.015	3.324
Adult	SE	Hi PV	Medial	9.339	1.695
			Lateral	31.221	3.915
		Lo PV	Medial	27.771	3.108
			Lateral	48.998	4.292
	EE	Hi PV	Medial	9.632	1.608
			Lateral	36.872	3.714
		Lo PV	Medial	46.414	2.948
			Lateral	60.983	4.071

*Means and standard error of the means (s.e.m.) (x10^–6^ cells/μm^2^) of PV cell densities presented based on age (P15 vs. adult), housing (SE vs. EE), cell type (Hi vs. Lo PV) and region (Medial vs. Lateral). All factors contributed to differences, with EE groups exhibiting significantly greater Lo PV cell densities in lateral regions at P15 as well as both medial and lateral areas as adults (see text for details).*

Further, when compared across regions, there was a significantly higher density of both Hi and Lo PV cells in lateral versus the medial striatum in P15 SE (Hi PV lateral > medial: *p* = 0.001; Lo PV lateral > medial: *p* = 0.011), EE (Hi PV lateral > medial: *p* < 0.001; Lo PV lateral > medial: *p* < 0.001), as well as adult SE (Hi PV lateral > medial: *p* < 0.001; Lo PV lateral > medial: *p* < 0.001) and EE (Hi PV lateral > medial: *p* < 0.001; Lo PV lateral > medial: *p* = 0.002) sections [Figure 2, black lines and crosses indicate significant differences across region related comparisons (same conventions used for subsequent figures)].

Across cell types, significantly greater densities of Lo vs. Hi PV cells were observed only in lateral striatum of EE cohorts at P15 [lateral Lo PV > Hi PV: *p* < 0.001; SE samples showed no differences at P15: *p* > 0.398; Figure 2, small black triangles indicate significance levels of comparisons across Hi vs. Lo PV cells depicted above and below the *x*-axis respectively (same conventions used for subsequent figures)]. By adulthood, however, Lo PV densities were higher in lateral as well as medial striatum for both SE (medial Lo PV > Hi PV: *p* < 0.001; lateral Lo PV > Hi PV: *p* = 0.014) and EE (medial Lo PV > Hi PV: *p* < 0.001; lateral Lo PV > Hi PV: *p* = 0.001) housing groups (Figure 2, black triangles).

Of primary interest here, comparisons across housing groups revealed that EE enhances PV expression at P15, but only for the Lo PV cells in the lateral striatum [EE > SE: *p* < 0.001; *p* > 0.20 for all other comparisons at this age; Figure 2, red lines with red asterisks indicate comparisons and significance levels between housing groups (same conventions used for subsequent figures)]. Housing-related effects persisted into adulthood, with Lo PV cells exhibiting significantly greater densities in EE subjects. By this stage significant differences were apparent for both medial (EE > SE: *p* < 0.001) and lateral regions of the nucleus (EE > SE: *p* = 0.049; Figure 2, red asterisks). Together, these findings suggest that EE has both a cell-type and region-specific influence on the emergence of PV expressing cells in the developing striatum.

### Environmental Enrichment Leads to Increased PNN Formation in Developing, but Not Adult Striatum

**FIGURE 3:**
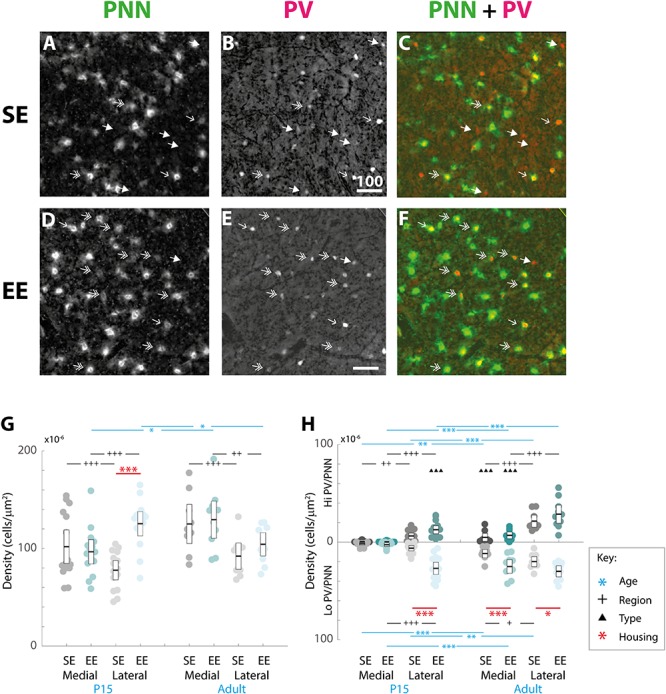
Age, region, cell type, and housing conditions affect PNN and PV/PNN overlap differently in the developing and adult striatum. **(A–F)** Images showing the pattern of PNN (WFA) staining **(A,C)**, PV expression **(B,E)**, and their overlap **(C,F)** in the lateral striatum of adult mice raised in standard (SE; top row) and enriched (EE: bottom row) housing. Both Hi PV (single-headed, unfilled arrows) and Lo PV (double headed arrows) cells are detectable in SE and EE samples. Unlike in cortex, only a partial overlap between PV cells and PNNs is observed in the striatum. Filled arrows indicate PV-positive cells that are not encapsulated by PNNs. Overall, PV cell density appears to have increased in the EE section (compare **E** to **B**), while little or no difference is observable for PNNs (compare **D** to **A**). Scale bar (in image): 100 μm. **(G)** Striatal PNN density (counts per micrometer^2^) in standard (SE) and enriched (EE) mice at all ages assessed. Conventions are identical to those in Figure 2. Blue bars and asterisks (Age): No differences in PNN densities by age were revealed in either region for SE samples (SE by age, *p* > 0.07 for medial and lateral domains). A significant increase in adult compared to P15 PNN levels was, however, detected in medial striatum in EE mice (Adult > P15: *p* = 0.010). Curiously, lateral densities exhibited a significant decrease with age in this cohort (P15 > Adult: *p* = 0.019). Black bars and crosses (Regions): Significant differences were observed between striatal regions, with medial densities greater than lateral for P15 SE samples (Medial > Lateral, *p* < 0.001). EE pups exhibited the opposite bias at this age point (Lateral > Medial, *p* < 0.001). For adult samples, both SE (*p* < 0.001) and EE (*p* = 0.002) groups exhibited greater medial PNN densities. Red bars and asterisks (Housing): At P15, significantly greater PNN densities were detected in EE relative to SE samples in lateral striatum (*p* < 0.001). Differences between housing groups, however, were not maintained in adult samples for either striatal region. Means (solid bar) and standard errors of the mean (s.e.m.; extent of white boxes) are depicted. Individual values are depicted as coloured circles: darker greys: medial SE; darker cyans: medial EE; lighter greys: lateral SE; lighter cyans: lateral EE. ^∗^*p* < 0.05; ^∗∗^*p* < 0.01; ^∗∗∗^*p* < 0.001; ^+^*p* < 0.05; ^++^*p* < 0.01; ^+++^*p* < 0.001. **(H)** Density (counts per micrometer^2^) of PV/PNN overlap in SE and EE mice at all age points assessed. Blue bars and asterisks (Age): Across ages, overlap with both Hi (Medial: *p* = 0.005; Lateral: *p* < 0.001) and Lo PV cells (Medial: *p* < 0.001; Lateral: *p* = 0.001) were significantly greater in adults compared to P15 mice, in both medial and lateral striatum for SE cohorts. In EE mice, both Hi PV/PNN (EE: Hi PV/PNN: *p* < 0.001) and Lo PV/PNN (Lo PV/PNN: Adults > P15, *p* < 0.001) overlap was significantly higher in adults compared to P15 samples in medial striatum. Significantly greater densities were also detected for adult Hi PV/PNN (*p* < 0.001) but not Lo PV/PNN (*p* = 0.403) associations in the lateral region. Black bars and crosses (Regions): Overlap in lateral compared to medial regions was significantly higher for Hi PV cells in both SE (*p* = 0.009) and EE samples (*p* < 0.001). Overlap densities in lateral striatum were greater for Lo PV cells only in EE samples at the same age (*p* < 0.001). A similar trend was observed in adult samples, with greater Hi PV/PNN associations detected in lateral compared to medial striatum for SE (*p* < 0.001) and EE (*p* < 0.001) groups. Regional differences in *(Lo PV/PNN overlap densities were detected in SE (Lateral > Medial: *p* = 0.018) but not EE mice (*p* = 0.124). Black triangles (Cell Types): No cell type differences between Hi and Lo PV/PNN overlap were revealed at P15 for SE striatum (*p* > 0.36). In EE samples, however, significantly greater Lo PV/PNN compared to Hi PV/PNN associations were observed in lateral (*p* < 0.001), but not medial (*p* = 0.088) striatum at this age. In adulthood, Lo PV/PNN overlap was detectably higher in medial, but not lateral portions of the nucleus for both SE and EE groups (Adult SE Medial, Lo PV/PNN > Hi PV/PNN: *p* < 0.001; EE Medial, Lo PV/PNN > Hi PV/PNN: *p* < 0.001; no differences in lateral striatum for either housing group at this age: *p* > 0.73). Red bars and asterisks (Housing): Significantly greater Lo PV/PNN (*p* < 0.001; black asterisks) overlap densities were detected in EE relative to SE samples in lateral striatum at P15. PV/PNN overlap exhibited significantly higher densities in both medial (*p* < 0.001; black asterisks) and lateral (*p* = 0.019; black asterisks) regions of EE sections in adults. No differences in Hi PV/PNN densities were observed. Means (solid bar) and standard errors of the mean (s.e.m.; extent of white boxes) are depicted. Conventions are identical to Figure 2. ^∗^*p* < 0.05; ^∗∗^*p* < 0.01; ^∗∗∗^*p* < 0.001; ^+^*p* < 0.05; ^++^*p* < 0.01; ^+++^*p* < 0.001; ^▲^*p* < 0.05; ^▲▲^*p* < 0.01; ^▲▲▲^*p* < 0.001.)*

Although EE has already been demonstrated to influence the initial emergence of striatal WFA labelled PNNs (Simonetti et al., 2009), the long-term effect on adult striatum has yet to be characterised. In order to determine whether enrichment had a continuing effect on the formation and maintenance of striatal PNNs, overall densities of these extracellular matrix structures within medial and lateral regions at P15 and adulthood were compared between housing groups (Figures 3A,D).

Omnibus testing revealed that only housing conditions (SE versus EE) affected PNN densities [MM ANOVA, between subject main factor of housing condition: *F*(1,45) = 4.847, *p* = 0.033]. A significant interaction was also detected, suggesting that EE had a differential effect on PNN formation depending on age and striatal region [housing ^∗^ age ^∗^ region: *F*(1,45) = 9.929, *p* = 0.003].

Overall developmental trajectories appeared to vary considerably between housing groups. While no significant age-related differences in PNN densities were revealed in either region for SE samples (SE by age, *p* > 0.07 for medial and lateral domains), a significant increase in PNN levels were detected in medial striatum in EE mice (EE by age, medial: Adult > P15, *p* = 0.010; Figure 3G, blue asterisks). Curiously, lateral PNN densities exhibited a significant decrease with age in this group (EE by age, medial: P15 > Adult, *p* = 0.019; relevant descriptive statistics can be found in Table 2; Figure 3G, blue asterisks).

**TABLE 2:** PNN densities.

**Age**	**Housing**	**Region**	**Mean**	**Standard Error**
P15	SE	Medial	101.866	7.694
		Lateral	77.747	5.463
	EE	Medial	96.660	7.694
		Lateral	125.348	5.463
Adult	SE	Medial	125.096	9.933
		Lateral	92.168	7.053
	EE	Medial	129.488	9.423
		Lateral	104.258	6.691

*Means and s.e.m.s (x10^–6^ cells/μm^2^) of PNN densities presented based on age (P15 vs. adult), housing (SE vs. EE), and region (Medial vs. Lateral). Densities varied with age, region, and housing group. Differences between EE and SE values were detectable only at P15 (see text for details).*

Significant differences were also observed between striatal regions, with medial densities greater than lateral for P15 SE pups (SE, P15 Medial > Lateral, *p* < 0.001, Figure 3G, black crosses). EE pups exhibited the opposite bias at this age point (EE, P15: Lateral > Medial, *p* < 0.001; Figure 3G, black crosses). For adult samples, both SE (Medial > Lateral, *p* < 0.001) and EE (Medial > Lateral, *p* = 0.002) housing groups exhibited greater medial PNN densities (Figure 3G, black crosses).

Pairwise comparisons between housing groups revealed significantly greater PNN densities in lateral striatum of EE compared to SE mice at P15 (P15 PNNs in lateral striatum, EE > SE: *p* < 0.001; Figure 3G, red asterisks). Housing-related effects did not persist into adulthood, however, with no detectable differences observed in either striatal region of adult samples (*p* > 0.200 for all comparisons). Together these findings suggest that enrichment is having a dynamic influence on striatal PNN formation and maintenance across the ages examined.

### Environmental Enrichment Leads to Increased PNN Encapsulation of PV Cell Populations in Developing and Adult Striatum

Although primary targets for PNN encapsulation in cortex (Pizzorusso et al., 2002), only around half of all PV cells are associated with PNNs in striatum (Lee et al., 2012). This is consistent with recent evidence suggesting that PV expression (Sugiyama et al., 2008) and PNN formation (Miyata et al., 2012) may be regulated by separate, but related, activity-dependent and/or independent processes. How EE influences the encapsulation of PV cells by PNNs, given the observed differences in striatal PV and PNN expression, has yet to be determined. Accordingly, we compared the densities of cells encapsulated by PNNs in the same SE and EE samples.

Qualitative examination of PNN formation and PV/PNN overlap confirms previous findings in SE mice (Figures 3A–F). Similar to PV expression, PNN encapsulation emerges over the first few weeks of postnatal development for both SE and EE samples (Lee et al., 2008). While a sizeable number of PV cells are encapsulated by these glycoprotein structures [Figures 3C,F; single (Hi PV) and double (Lo PV) arrows] the relationship is not exclusive (Figures 3C,F; PV cells without PNN encapsulation represented by filled arrows; see also Lee et al., 2012). Percentages of total PV cells encapsulated by PNNs in adult samples were similar to values previously reported (Table 3, see also Lee et al., 2012). Although overall PV density appears to be greater for EE sections (compare Figure 3E to Figure 3B), PNN density seem to exhibit little difference (compare Figure 3D to Figure 3A; see above).

**TABLE 3:** Percentages of PV cells encapsulated by PNNs.

**Age**	**Housing**	**Region**	**Mean**	**Standard Error**
P15	SE	Medial	44.306	6.893
		Lateral	47.715	4.563
	EE	Medial	47.378	6.622
		Lateral	72.450	4.384
Adult	SE	Medial	41.822	7.959
		Lateral	52.145	5.268
	EE	Medial	55.654	7.551
		Lateral	59.180	4.998

*Means and s.e.m.s of PNN encapsulated over PV total percentages [(Overlap/PV totals) ^∗^ 100] presented based on age (P15 vs. adult), housing (SE vs. EE), and region (Medial vs. Lateral). EE samples generally exhibited greater percentages of PV cells encapsulated by PNNs, particularly in the lateral region at both ages examined.*

When considering the effect of housing conditions (SE vs. EE), age (P15, and adult), cell type (Hi vs. Lo), and region (lateral vs. medial striatum) quantitatively, omnibus testing confirmed that densities of PNNs encapsulating PV cells were significantly affected by all factors [MM ANOVA, between subject main factor of housing condition: *F*(1,45) = 34.165, *p* < 0.001; age: *F*(1,45) = 78.737, *p* < 0.001; within subject factors for cell type: *F*(1,45) = 24.105, *p* < 0.001; and striatal region: *F*(1,45) = 252.451, *p* < 0.001]. A significant interaction was also detected, suggesting that housing condition influenced PV/PNN associations differently depending on cell type, age, and striatal region [housing ^∗^ age ^∗^ cell type ^∗^ region: *F*(1,45) = 5.922, *p* = 0.019].

Developmental trajectories of PV/PNN overlapped broadly with PV expression. Age saw an increase in PV/PNN associations for Hi and Lo PV cell types in SE medial (Hi PV/PNN Adults > P15, *p* = 0.005; Lo PV/PNN Adults > P15, *p* < 0.001) lateral (Hi PV/PNN Adults > P15: *p* < 0.001; Lo PV/PNN Adults > P15: *p* = 0.001), as well as EE medial (Hi PV/PNN Adults > P15, *p* < 0.001; Lo PV/PNN Adults > P15, *p* < 0.001) striatal regions. Hi PV/PNN (Adult > P15: *p* < 0.001), but not Lo PV/PNN (Adult > P15, *p* = 0.403) overlap also exhibited age dependent increases in EE lateral striatum (relevant descriptive statistics can be found in Table 4; Figure 3H, blue asterisks).

**TABLE 4:** PV/PNN overlap densities.

**Age**	**Housing**	**Type**	**Region**	**Mean**	**Standard Error**
P15	SE	Hi PV	Medial	0.497	0.918
			Lateral	6.346	2.410
		Lo PV	Medial	1.735	1.549
			Lateral	6.206	2.295
	EE	Hi PV	Medial	0.330	0.918
			Lateral	12.702	2.410
		Lo PV	Medial	2.678	1.549
			Lateral	26.853	2.295
Adult	SE	Hi PV	Medial	4.934	1.185
			Lateral	21.523	3.112
		Lo PV	Medial	11.724	2.000
			Lateral	19.969	2.963
	EE	Hi PV	Medial	6.987	1.124
			Lateral	28.715	2.952
		Lo PV	Medial	24.919	1.897
			Lateral	29.920	2.811

*Means and s.e.m.s (x10^–6^ cells/μm^2^) of PV/PNN overlap densities presented based on age (P15 vs. adult), housing (SE vs. EE), cell type (Hi vs. Lo PV) and region (Medial vs. Lateral). Overlap densities varied with all factors. EE samples exhibited significantly greater Lo PV/PNN densities in lateral regions at P15. Both SE as well as EE adults revealed greater Lo PV/PNN overlap in medial, but not lateral areas (see text for details).*

When considering regional biases, greater Hi PV/PNN overlap densities were detected in lateral compared to medial domains for SE (Lateral > Medial: *p* = 0.009) and EE (Lateral > Medial: *p* < 0.001) housing groups at P15. Lo PV/PNN overlap was higher in the lateral domain only for EE pups (Lateral > Medial: *p* < 0.001; Figure 3H, black crosses). A lateral bias persisted for both SE (Lateral > Medial: *p* < 0.001) and EE (Lateral > Medial: *p* < 0.001) housing groups in adult Hi PV/PNN overlap (Figure 3H, black crosses). For Lo PV/PNN, however, greater lateral densities could only be detected in SE mice (Adult SE Lo PV Lateral > Medial: *p* = 0.018; Lo PV lateral not different from Medial for EE samples: *p* = 0.124; Figure 3H, black crosses).

Significantly greater densities of Lo vs. Hi PV/PNN overlap were observed in lateral (Lo PV/PNN > Hi PV/PNN: *p* < 0.001) but not medial (Lo PV/PNN > Hi PV/PNN: *p* = 0.088) striatum of EE samples at P15 (*p* > 0.36 for all SE comparisons at this age; Figure 3H, black triangles). By adulthood, Lo PV densities were higher in medial striatum for SE (Lo PV/PNN > Hi PV/PNN: *p* < 0.001) and EE (Lo PV/PNN > Hi PV/PNN: *p* < 0.001) mice; no differences in PV/PNN overlap with respect to cell type were observed for either group in the lateral region (*p* > 0.73 for both housing groups; Figure 3H, black triangles).

Comparing PV/PNN overlap between housing groups confirmed a strong effect of enrichment. Significantly greater PNN overlap with Lo PV cells was observed in the lateral striatum of EE compared to SE mice at P15 (Lo PV/PNN Lateral, EE > SE: *p* < 0.001; all other comparisons: *p* > 0.06 for this age; Figure 3H, red asterisks). Housing related effects persisted into adulthood, with detectably higher Lo PV/PNN overlap observed in both medial and lateral striatum of adult EE cohorts (Lo PV/PNN Medial, EE > SE: *p* < 0.001; Lo PV/PNN Lateral, EE > SE: *p* = 0.019; Figure 3H, red asterisks). These findings suggest that the effects of EE on PNN encapsulation of PV cells may be related to a similar influence enrichment has on emergent PV expression.

### Enrichment Has a Transient Effect on Striatal Brain-Derived Neurotrophic Factor Levels in Postnatal Developing Mice

Accelerated expression of BDNF in enriched mice has been shown to correlate with earlier maturation of inhibitory interneurons within cortex (Huang et al., 1999; Cancedda et al., 2004; Sale et al., 2004). To determine whether a similar link between trophic factor expression and interneuron maturation as measured by PV expression is present in the striatum, we compared the presence of BDNF protein within the nucleus of enriched and standard housed pups at three early postnatal ages: P8, P10, and P15, time-points just prior to, and inclusive of the early stages of PV expression.

**FIGURE 4:**
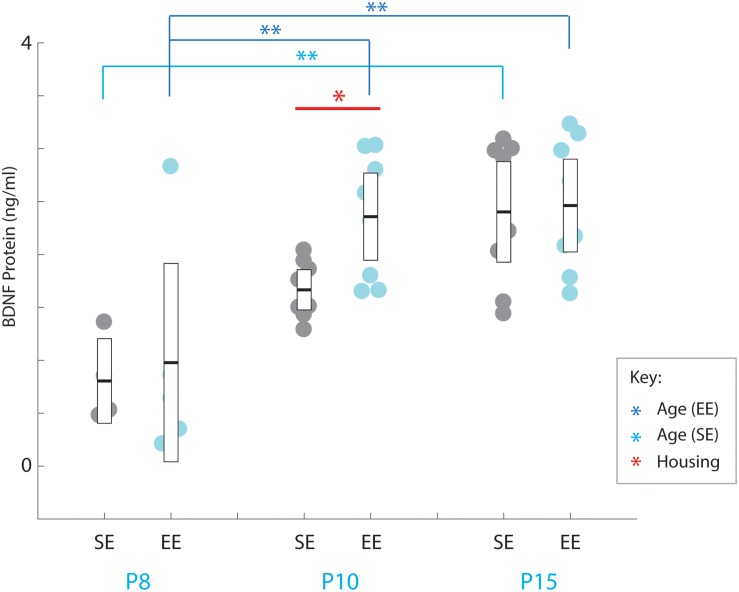
Environmental enrichment accelerates the increase in BDNF protein levels in early postnatal striatum. BDNF protein concentrations (ng/ml) for individual standard (SE; grey), and enriched (EE; cyan) housed mice at ages P8 (*n* = 5), P10 (*n* = 8), and P15 (*n* = 8). Dark blue brackets and asterisks (EE Samples): EE samples exhibited significantly higher concentrations of BDNF at P10 and P15 compared to P8 levels (P10: *p* = 0.002; P15: *p* = 0.001). Light blue bracket and asterisks (SE Samples): SE material only yielded differences between P8 and P15 (*p* = 0.001). Red bar and asterisks (Housing): Pairwise comparisons revealed a significantly greater level of BDNF protein present in the striatum of EE vs. SE mice at P10 (*p* = 0.036; black asterisk). Means (solid bar) and standard errors of the mean (s.e.m.; extent of white boxes) are depicted. ^∗^*p* < 0.05; ^∗∗^*p* < 0.01.

A strong overall effect of age was detected across the postnatal period assessed [Age: *F*(2,35) = 17.190, *p* < 0.001; Figure 4]. Pair-wise comparisons revealed that P8 BDNF levels were significantly less than all other ages probed for EE mice (P8 vs. P10: *p* = 0.002; P8 vs. P15: *p* = 0.001; P10 vs. P15: *p* = 1.000; relevant descriptive statistics can be found in Table 5; Figure 4, dark blue brackets and asterisks: brackets show age groups that were significantly different indicated by asterisks), while only a difference was observed between P8 and P15 for SE subjects (P8 vs. P10: *p* = 0.096; P8 vs. P15: *p* = 0.001; P10 vs. P15: *p* = 0.078; Figure 4, light blue brackets and asterisks indicate significant differences). These findings suggest that the presence of the trophic factor increases reliably during the second postnatal week in the developing striatum.

**TABLE 5:** BDNF protein levels.

**Housing**	**Age**	**Mean**	**Standard Error**
SE	P8	0.804	0.316
	P10	1.668	0.224
	P15	2.403	0.224
EE	P8	0.977	0.283
	P10	2.358	0.224
	P15	2.461	0.224

*Means + s.e.m.s (ng/ml) of BDNF protein levels presented based on housing group and age. BDNF exhibited significant increases with age for both housing groups. A small increase in EE compared to SE samples was also detected at P10 (see text for details).*

While no overall effect due to enrichment was observed [Housing condition: *F*(1,35) = 2.239, *p* = 0.143; housing condition ^∗^ age interaction: *F*(2,35) = 1.084, p = 0.349], pairwise comparisons did reveal a detectable difference between SE and EE BDNF levels at P10 (P10 SE vs. EE: *p* = 0.036; Figure 4, red asterisk). While only preliminary, this finding suggests the possibility that enrichment from birth may expedite the postnatal increase in BDNF, potentially contributing to the subsequent dramatic increase in PV expression observed in P15 EE mice.

## Discussion

This study provides further evidence that early EE is capable of dramatically affecting postnatal striatal development. Enrichment led to significant increases in striatal PV expression at P15 and into adulthood. Moreover, the influence of EE is cell type and region specific: significantly greater densities were primarily detectable for Lo PV populations, with differences first observed within lateral striatum and spreading medially with age, following the normal spatiotemporal pattern of PV expression within the nucleus (Schlosser et al., 1999).

EE also had an effect on striatal WFA labelled PNN density, although housing related changes were only observed within the lateral striatum at P15. The influence of enrichment on PV cell encapsulation by PNNs generally followed PV expression in terms of regional and cell type differences. Given the lack of detectable differences between housing groups in adult striatal PNN densities, this finding suggests that at least some of the non-PV expressing cells encapsulated by PNNs in SE mice may actually be capable of expressing PV under appropriate conditions. Finally, a small, but nevertheless significant increase was observed in striatal BDNF levels of EE samples at P10, suggesting a possible mechanism underlying the heightened PV expression detected in enriched mice.

### Technical Limitations

The enriched cages used in this study provided enhanced stimulation of sensory modalities as well as increased opportunities for voluntary exercise and social interaction. While this approach resulted in detectable differences in the expression of markers associated with circuit maturation in the striatum, the nucleus itself is involved in regulating, updating, and maintaining processes (e.g., the acquisition of motor skills; the formation of sensori-motor associations; the acquisition of goal directed behaviours) that involve all of these factors in a region specific manner (Yin et al., 2004, 2006). Based on our means of enrichment, it is not possible to determine which specific feature of the enhanced home cages most contributed to the changes observed across the mediolateral extent of the striatum in EE mice.

Further, although a rigorous assessment of cage activity was beyond the scope of this study, passive observation during routine monitoring indicated that mice actively engaged in the larger and more dynamic environment. Moreover, when assessed directly, we have previously shown that exposure to this EE paradigm from birth leads to increased exploratory behaviour in developing mice, as well as improved task acquisition and performance (Simonetti et al., 2009; O’Connor et al., 2014; Rountree-Harrison et al., 2018). Experiments that differentiate specific features of enriched environments (e.g., social, sensory, voluntary exercise) will be required to determine how they contribute to the changes observed in striatal circuitry of enriched pups.

PNNs are formed via an aggregated assembly of hyaluronan, tenascin-R, and chondroitin sulphate proteoglycans that can include aggrecan, brevican, neurocan, and others (Oohira et al., 1994; Giamanco et al., 2010; Giamanco and Matthews, 2012; Dauth et al., 2016; Favuzzi et al., 2017; Yamada and Jinno, 2017). These constituents vary depending on brain area. WFA is a lectin that binds to the glycosaminoglycan (GAG) chains of CSPGs (Pizzorusso et al., 2002). Thus, while WFA can reliably report the presence of CSPGs in PNNs (Bruckner et al., 2000; Pizzorusso et al., 2002), it cannot identify the types of proteoglycans linked within the structure. Further studies will be required to determine the exact composition of striatal PNNs and if and how this may vary with age and EE.

Recent work has revealed that male and female mice exhibit different expression levels of Foxp2, a key regulator of ultrasonic vocalisation, in the developing striatum (Frohlich et al., 2017). Further, enrichment has been shown to induce sex linked effects on the elevated plus maze performance (Hendershott et al., 2016). While samples from both males and females were included in this study, as revealing sex related differences was not a part of our original experimental design, once divided by gender, the relatively small numbers of sections available for analysis meant that no reliable conclusions could be made regarding the influence of this factor. It would be of interest to determine whether the manner in which EE influences striatal PV and PNN development differs in males and females, given the critical role the nucleus plays in potentially sexually dimorphic sensorimotor learning and goal directed behaviour.

Finally, BDNF expression levels were examined at time points just prior to, and inclusive of P15, the first age explored in this study. While our findings support a possible link between EE-induced increases in neurotrophic and PV expression, it should be noted these are correlative only. A more exhaustive study that explicitly assesses BDNF levels in different striatal regions across a broader range of ages would be required to determine a definitive relationship between the two.

### Accelerated Maturation of Parvalbumin-Expressing Inhibitory Circuitry in Enriched Pups

In rats, striatal PV expression was reported to emerge between the first and second postnatal weeks, exhibiting a lateral to medial developmental trajectory (Schlosser et al., 1999), with a lateral bias in PV cell density maintained into adulthood. In this study, a similar overall patterning was observed in murine striatal PV distributions for both housing groups, with expression emerging within the lateral striatum first, maintaining significantly higher levels in this region as mice reach adulthood.

The striatum can be divided into regions based on anatomical connectivity as well as function. The dorsomedial region, which is the recipient of input from prefrontal, entorhinal, and higher order sensory cortical areas (Hintiryan et al., 2016), is involved in the acquisition of goal-directed action-outcome associations (Yin et al., 2005a, b). The dorsolateral region on the other hand, receives topographically aligned input from primary sensory as well as motor areas (Hintiryan et al., 2016), and is involved in sensorimotor learning, movement/action coordination, and habit formation (Yin et al., 2004, 2006). These sources appear to differ in terms of corticofugal cell distributions as well as terminal patterning, with lateral striatum-targetting sensorimotor cortex tending to have greater densities of projecting neurons with smaller terminal fields, potentially affecting the degree of convergence onto, and activation of input recipient neurons, including PV cells (Bennett and Bolam, 1994). Mechanistically, excitatory synaptic release of BDNF and other factors such as the homeobox protein Otx-2 have been shown to contribute to emergent PV expression (Sugiyama et al., 2008; Beurdeley et al., 2012; Spatazza et al., 2013). While the specific patterning of corticostriatal input and activation on individual cells has yet to be determined, our findings suggest that differences in network level connectivity may be contributing to the persistent lateral bias in striatal PV density.

The importance of cortical excitatory drive is also reflected in the developmental trajectory of striatal PV expression. Prefrontal areas tend to mature later than sensory cortex (Bruckner et al., 2000; Baker et al., 2017). The later onset of PV expression in medial striatum may therefore be due, at least in part, to the delayed postnatal developmental trajectories of cortical areas projecting to this region.

Both the earlier-generated Hi PV and later generated Lo PV populations of interneurons are present in the striatum (Donato et al., 2015). These cell types have been shown previously to differ in terms of function, with Hi PV cells involved in consolidating already acquired information, and Lo PV cells preferentially activated during the learning process (Donato et al., 2015). In hippocampus, the relative abundance of Lo PV cells is increased by EE, contributing to the heightened plasticity associated with enrichment (Donato et al., 2013). In the striatum, EE was able to significantly increase the density of Lo PV cells at P15 and into adulthood, suggesting enrichment exerted a similar effect on striatal PV expression. The larger enclosure, combined with increased opportunities for sensory stimulation, social interactions, and voluntary exercise means that the relative opportunity for acquiring novel associations is far greater in enriched housing conditions. Accordingly, in as much as the transition from Lo to Hi PV expression in new born cells is dynamic (Donato et al., 2013, 2015), the increase in striatal Lo PV cell density may reflect a cumulative difference in the extent of novel experiences encountered by SE and EE mice. It remains to be seen whether the actual ability to acquire information differs between housing groups over and above the putative cumulative effects of home cage exposure.

### Environmental Enrichment Affects PNN Formation in Developing Striatum

Striatal PNN formation was also affected during postnatal development in EE samples, consistent with previous findings (Simonetti et al., 2009), albeit in a region specific manner. Unlike PV expression, however, housing-related effects did not persist into adulthood.

PNN formation is dependent on a complex interplay between activity-dependent (Pizzorusso et al., 2002) and independent processes (Gati et al., 2010). A shift in sulphation from C6 to C4 of chondroitin sulphate chains, a developmental programme that appears to not require neural activation *per se*, has been shown to drive initial PNN condensation (Miyata et al., 2012). Consistent with this, the initial emergence of striatal PNN formation is not noticeably affected by enrichment (Simonetti et al., 2009).

On the other hand, activity-dependent factors have been shown to also influence PNN formation (Pizzorusso et al., 2002; Carulli et al., 2010). The significant lateral bias seen at P15 for EE striatum is broadly consistent with the enrichment-induced changes observed in PV expression.

The significant decrease in lateral PNN expression of EE samples from adults compared to P15, however, was unexpected. Recent work has revealed that EE can have a profound, if different effect on adult versus developing cortex. While enrichment can accelerate PNN formation in developmental critical periods (Ciucci et al., 2007; Simonetti et al., 2009), EE can have a depleting effect on the overall density of these structures found in mature cortex (Sale et al., 2007). It may be that the decrease in lateral PNN densities of mature EE striatum is reflective of a similar change in the influence of enrichment across the lifetime of the animal. Further work will be required to assess this possibility.

### Environmental Enrichment From Birth Influences Overlap Between Striatal Parvalbumin and PNN Expression in Adult Mice

Overall, enrichment affected the degree of PV/PNN overlap, increasing the density of encapsulated cells. This effect generally followed the developmental trajectory of emergent PV expression, initiating within the lateral region of the nucleus at P15, and encompassing both halves of the adult striatum, particularly for Lo PV cells.

In the striatum of standard-housed mice, only around half of PNNs are associated with PV expressing neurons (Lee et al., 2012). The identity of these non-PV cells associated with these glycoprotein structures has yet to be determined. Our finding of EE-dependent increases in overall overlap densities suggests the possibility that at least some of these PNNs are condensing onto cells that can be induced to express PV under enrichment conditions.

Latent PV expression is likely regulated by activity-dependent processes. Synaptic signalling molecules such as neuronal activity-regulated pentraxin (NARP) (Chang et al., 2010) require the presence of intact proteoglycans that provide scaffolding for PNNs to accumulate onto target cells. NARP is critical for activity dependent accumulation of excitatory drive onto PV expressing interneurons, a key step in recruiting inhibition and regulating network level plasticity associated with ‘critical periods’ in developing cortex (Gu et al., 2013). Given the likely increase in excitatory tone associated with having been raised in, and continually interacting within enriched environments, cortical afferents of EE mice targetting proteoglycan linked (potential) striatal PV cells may heighten the accumulation of NARP onto these interneurons, facilitating their maturation, as reflected by PV expression. Further work will be required to determine if this is the case.

### Postnatal Changes in BDNF Levels May Contribute to Emergent PV Expression

Preliminary protein analysis revealed a significant increase in BDNF levels between P8 and P10, prior to the noted differences in PV and PNN expression due to enrichment observed in this study (see also Simonetti et al., 2009, p. 270). Previous work in the developing visual system has revealed that transgenically driven increases in early BDNF levels expedite PV network maturation (Huang et al., 1999). BDNF, like PV expression has been shown to be accelerated with EE (Cancedda et al., 2004). Thus, the earlier increase in BDNF levels, prior to the observed changes in PV expression, observed in EE samples raises the possibility that a similar relationship between trophic factor and inhibitory networks may be present in the developing striatum. Whether and to what degree this is the case has yet to be determined.

Previous studies have revealed that BDNF protein is present within the lateral ganglionic eminence and striatal regions in the late embryonic period (Baydyuk et al., 2013). The neurotrophin is thought to be transported via mesencephalic DA neurons whose projections to the nucleus are present prenatally (Baydyuk et al., 2013). These findings implicate a crucial role for BDNF in development of medium spiny neurons (MSNs), the main output cells within the striatum.

While MSNs are present within the striatum before birth, PV expression does not begin until the 2nd postnatal week (Schlosser et al., 1999). Despite this, BDNF has been directly implicated in the proper maturation of PV positive cells (Altar et al., 1997). The timing of this emergence suggests either another event (e.g., Otx2, Sugiyama et al., 2008), or possibly a second source of trophic input may be required for the maturation of PV cells to initiate. In cortex, the maturation of inhibitory circuitry has been shown to be dependent on BDNF delivery via excitatory glutamatergic synaptic drive (Huang et al., 1999). As excitatory input to the striatum is sourced almost exclusively from exogenous cortical and related regions (Hintiryan et al., 2016), projections from these areas have the potential to augment striatal BDNF levels from the first postnatal weeks onward (Nisenbaum et al., 1998). Consistent with this, deafferentation of cortical input in adult rats leads to decreased PV expression within the nucleus (Altar et al., 1997). Thus, it is conceivable that the coordinated delivery of the trophic factor from both corticostriatal and nigrostriatal drive may be required to allow for the proper maturation of PV cells. As enrichment induces precocious, augmented development of cortical circuitry (Huang et al., 1999), this may in turn affect the delivery of BDNF to the striatum. Further experiments would be required to assess this possibility rigorously.

## Conclusion

Striatal PV fast spiking interneurons constitute fewer than 3% of neurons within the striatum (Kita et al., 1990; Kawaguchi, 1993). Although small in number, these gap-junction linked (Kita et al., 1990; Fukuda, 2009) recipients of direct cortical and nigral input (Bennett and Bolam, 1994; Plotkin et al., 2005), provide strong feed-forward inhibition to multiple proximal MSNs (Koos and Tepper, 1999; Tepper et al., 2004), suggesting an important role in regulating striatal output. Knowledge of how regional differences in PV cell expression and PNN encapsulation develop and persist in the murine striatum, and the manner in which EE affects these processes, may help us to understand how subtle differences in wiring can differentially influence circuit formation and function, as well as an awareness of how distinct but related processes are represented and influenced by environmental factors.

## Data Availability Statement

The datasets generated for this study are available on request to the corresponding author.

## Ethics Statement

All procedures were approved by the Animal Ethics Committee of the University of Sydney and conformed to National Health and Medical Research Council of Australia guidelines.

## Author Contributions

AO’C, CL, and AS conceived and designed the study. AO’C, GH, and HM collected the data and generated the images. AO’C, TB, GH, CL, and AS organised and performed the preliminary analyses on the data. TB and AS performed the statistical analyses. AO’C, CL, and AS wrote the manuscript. All authors contributed to manuscript revision, and read and approved the submitted version.

## Conflict of Interest

The authors declare that the research was conducted in the absence of any commercial or financial relationships that could be construed as a potential conflict of interest.
